# Breast milk n-3 long-chain polyunsaturated fatty acids and blood pressure: an individual participant meta-analysis

**DOI:** 10.1007/s00394-020-02310-4

**Published:** 2020-06-20

**Authors:** Lenie van Rossem, Henriette A. Smit, Martine Armand, Jonathan Y. Bernard, Hans Bisgaard, Klaus Bønnelykke, Signe Bruun, Barbara Heude, Steffen Husby, Henriette B. Kyhl, Kim F. Michaelsen, Ken D. Stark, Carel Thijs, Rebecca K. Vinding, Alet H. Wijga, Lotte Lauritzen

**Affiliations:** 1 Julius Center for Health Sciences and Primary Care, University Medical Center Utrecht, Utrecht University, Universiteitsweg 100, 3584 GA Utrecht, The Netherlands; 2 Department of Obstetrics and Gynaecology, Erasmus MC University Medical Center, Rotterdam, The Netherlands; 3 Aix Marseille Univ, CNRS, CRMBM, Marseille, France; 4 Université de Paris, Centre for Research in Epidemiology and StatisticS (CRESS), INSERM, INRA, 75004 Paris, France; 5 COPSAC, Copenhagen Prospective Studies on Asthma in Childhood, Herlev and Gentofte Hospital, University of Copenhagen, Copenhagen, Denmark; 6 Strategic Business Unit Pediatric, Arla Foods Ingredients Group P/S, Viby J, Denmark; 7 Hans Christian Andersen Children’s Hospital, Odense University Hospital, Odense, Denmark; 8 Department of Clinical Research, Faculty of Health Sciences, University of Southern Denmark, Odense, Denmark; 9 OPEN, Odense Patient Data Explorative Network, Odense University Hospital, Odense, Denmark; 10 Department of Nutrition, Exercise and Sports, University of Copenhagen, Copenhagen, Denmark; 11 Department of Kinesiology, Faculty of Applied Health Sciences, University of Waterloo, Waterloo, Canada; 12 Department of Epidemiology, Care and Public Health Research Institute (CAPHRI), Maastricht University, Maastricht, The Netherlands; 13 Center for Nutrition, Prevention, and Health Services, National Institute for Public Health and the Environment, Bilthoven, The Netherlands

**Keywords:** Breastfeeding, Blood pressure, Fatty acids, Children

## Abstract

**Purpose:**

It is controversial whether a higher intake of n-3 long-chain polyunsaturated fatty acids (n-3 LC PUFA) through breastfeeding is associated or not to a lower blood pressure (BP) during childhood. We aimed to clarify this point by undertaking a meta-analysis involving the data from seven European birth cohorts.

**Methods:**

We searched https://www.birthcohort.net for studies that had collected breast milk samples, and had at least one BP measurement in childhood. Principal investigators were contacted, and all agreed to share data. One additional study was identified by contacts with the principal investigators. For each cohort, we analyzed the association of breast milk n-3 LC PUFAs with systolic and diastolic BP with linear mixed effects models or linear regression, and pooled the estimates with a random effects model. We also investigated age-specific and sex-specific associations.

**Results:**

A total of 2188 participants from 7 cohorts were included. Overall, no associations between breast milk n-3 LC PUFAs and BP were observed. In the pooled analysis, each 0.1 wt% increment in breast milk docosahexaenoic acid (DHA) was associated with a 1.19 (95% CI − 3.31, 0.94) mmHg lower systolic BP. Associations were similar for boys and girls and at different ages.

**Conclusion:**

In this individual participant meta-analysis, we found no evidence for an association between breast milk n-3 LC PUFAs and BP.

**Electronic supplementary material:**

The online version of this article (10.1007/s00394-020-02310-4) contains supplementary material, which is available to authorized users.

## Introduction

Breastfeeding has established short term advantages for the infants such as fewer infections [1], but may benefit longer term health as well, especially on cardiovascular health. Indeed, three meta-analyses reported associations of breastfeeding as compared with infant formula with lower blood pressure (BP) in childhood and adulthood [2–4].

The composition of breast milk strongly differs from infant formula, among others as breastmilk contains n-3 long-chain polyunsaturated fatty acids (n-3 LCPUFAs), especially EPA (eicosapentaenoic acid) and DHA (docosahexaenoic acid), which traditionally were not present in infant formula [5]. The beneficial effect of breastfeeding as compared to infant formula on BP is suggested to be due to differences in n-3 LC PUFA, especially DHA. N-3 LC PUFAs are important for BP control, among others via incorporation in endothelial cells in blood vessels, effects on balance between sympatic and parasympatic signaling from the brain, and eicosanoid regulation of BP [6–8]. In addition, there is a competition in the biosynthesis of DHA and arachidonic acid (AA), a n-6 LCPUFA, as the same enzymes are involved in the conversion steps from their respective precursors alpha-linolenic acid (n-3 PUFA) or linoleic acid (n-6 PUFA) [9]. This potential mechanism could be especially important in early life, where exposure may permanently change the function and structure of organs [10]. In addition, DHA deficiency in the perinatal period has been shown to be associated with an alteration in BP control later in life in rats [11]. A simple comparison of breastfeeding with formula feeding can not fully elucidate the potential role of n-3 LC PUFAs, as the fatty acid composition of breast milk varies between mothers [12], due to maternal diet and metabolic status [13, 14] and maternal genes [15].

Previous studies on the role of n-3 LC PUFAs in healthy infants born at term on BP later in life reported mixed findings, but the studies differ widely in methods of exposure and age of BP assessment in the children. One longitudinal cohort study observed that breast milk with a relatively high content of n-3 LC PUFA associated with a lower BP in children at 12 years of age when EPA and DHA levels were at or more than median values, i.e. 0.05% for EPA and 0.18% for DHA [16]. Randomized trials have examined effects of enriched infant formula [17, 18] or direct fish oil supplementation of infants [19] as well as n-3 LC PUFA supplementation of lactating mothers [20–23]. In the latter case, BP was not different at the age of 2.5 years, but BP was higher in boys at the age of 7 years and 13 years of age in those whose mothers received supplementation during lactation.

In the present study, we investigated the association between n-3 LC PUFAs in breast milk and long term BP with a meta-analysis using individual participant data (IPD) from seven birth cohort studies. Furthermore, we examine potential age-specific and sex-specific associations. Earlier studies suggested that DHA (22:6n-3) is the most important of the n-3 LC PUFAs regarding its effect on BP in adults [24, 25]; this was also observed in the previous study on breast milk fatty acids and BP [16]. Therefore, our primary interest was in DHA. We also assessed eicosapentaenoic acid (EPA, 20:5n-3) and considered the ratio of DHA/AA.

## Methods

### Participating cohorts and design

We performed an individual participant data (IPD) meta-analysis. Using the https://www.birthcohorts.net website, we identified six studies that had collected breast milk samples and had at least one measurement of BP during childhood. All six eligible birth cohorts (COPSAC2000 [26], COPSAC2010 [27], EDEN [28], KOALA [29], OCC [30], PIAMA [31]) agreed to participate. The principal investigators of the studies or their representatives were invited to share data on breast milk fatty acid composition, BP, and covariates of their study. Through the PIs of the earlier mentioned cohorts, one additional study (CU Trial) [32] was identified. This was an intervention study designed to supplement lactating mothers with fish oil, which assessed the resulting effect on breast milk fatty acid composition. The supplement in this trial provided 1.5 g/day of n-3 LC PUFA, which resembled the upper range in the intake from dietary sources in the Danish population. Other studies were observational in nature, or with an intervention that was unrelated to this research question (e.g. mattress covers in PIAMA for prevention of allergies), with the exception of COPSAC2010. In this study, women were randomized to supplementation during the third trimester of pregnancy with either 2.4 g/day fish oil (55% EPA and 37% DHA) or olive oil. Thus, we included seven studies from Denmark, France, and the Netherlands representing a total of 2188 participants. All studies obtained ethical approval from local institutional review boards and have therefore been performed in accordance with the ethical standards laid down in the 1964 Declaration of Helsinki and its later amendments. The data for this meta-analysis were stored in a single center on a secured data server with access for the first author (LvR) only.

### Breast milk fatty acid composition

Gas chromatography with flame ionization was used to determine the fatty acid composition of all the milk samples, although there were some differences in sampling, preparation and fatty acid analysis (please see Supplemental Table 1 for details). In general, these methods can produce relatively similar results although the use of acid-based catalysts can result in higher fatty acid recoveries [33], although at an increased risk of degrading of conjugated linoleic acid (a fatty acid of interest in dairy products) [34]. Expressing the fatty acid data as a relative weight percentage (wt%) of the total fatty acid content helps standardize fatty acid data [35], and we focused on DHA and EPA levels as well as DHA/AA ratio and not conjugated linoleic acid.

### BP measurements

The outcomes of interest were Systolic blood pressure (SBP) and Diastolic blood pressure (DBP). All studies used an automated oscillatory device to measure BP. The specific devices of each cohort are listed in Supplementary Table 1 of Online resource 1. Protocols were fairly similar, including a 5–10 min rest before measurements, an appropriately adjusted cuff size, measurement on the non-dominant or left arm, and a sitting or supine position. Furthermore, most studies (expect one) took several measurements which were averaged (with one study excluding the first measurement). We used BP in mmHg in the main analyses, adjusting for height, age, and sex in each analysis as BP in children is a function of length/height, age, and sex. In addition, for descriptive purposes, we show BP *z*-scores to be able to compare BP across ages. *z*-scores were derived from the Pediatric Hypertension Guidelines, updated from The Fourth Report on the Diagnosis, Evaluation, and Treatment of High BP in Children and Adolescents [36].

### Covariates

All cohorts obtained information on covariates; most covariates were based on self-reported questionnaires and some covariates were derived from medical records. These covariates were maternal age (years), maternal educational level (low, medium, high), maternal place of birth (European, non-European), maternal pre-pregnancy body mass index (BMI) (kg/m^2^), smoking status during pregnancy (yes, no), infant’s birth weight (grams), and gestational age (weeks) as the main potential confounders in the statistical analysis. All covariates were included a priori in the model, because of their known association with blood pressure.

### Statistical analysis

For each study, we describe the mean (SD) of breast milk fatty acid levels (wt%) and BP (mmHg and *z*-scores).

As the included studies measured BP at different ages, we assumed that statistical heterogeneity was present. Therefore, we applied a random effects meta-analysis which estimates the average association between the determinant and the outcome [37, 38]. We performed a two-stage meta-analysis. In the first stage, we ran two models for each study. Model 1 included the association between fatty acid level (DHA, EPA, or the DHA/AA ratio) and BP, adjusted for age, sex, and height. For this model, we fitted several models for height, by adding “height^2^” and “height^3^” to the model, and ran the model with the best fit for height, as determined by the -2log likelihood. For most studies, height was used as a single term. Six out of the seven included studies had repeated measurements of BP. For these studies a mixed model was used, to account for the intra-cohort correlations between the measurements. For the study with the single outcome measurement (i.e. KOALA), we used a linear regression model. In addition to height, age and sex, model 2 was further adjusted for the above-mentioned potential confounders and breastfeeding duration. In the second stage, the estimates of each study were pooled with a mixed model, where each cohort was a random effect, and each cohort was assigned a weighting factor (1/variance). Estimates represent the difference in BP (mmHg) per 0.1 wt% increase in breast milk fatty acids levels, which would be a realistic increase given the range of fatty acids in the studies.

From studies in adults on the association between fatty acids and BP, it has been suggested that the association may not show a dose–response relationship [39]. Therefore, we also examined associations with BP with breast milk DHA dichotomized at the median for each study.

We examined the interactions between breast milk and child sex and age in relation with BP by adding the appropriate interaction terms (e.g. DHA × sex) into the models.

As child’s height and child’s BMI could be an intermediate or modifying factor in the association between DHA and BP, given evidence in the literature on an association between DHA and height [40, 41] and DHA and BP in overweight children [42], we performed a sensitivity analysis where we used height and BMI as the outcome instead of BP. For those studies that had an intervention component by design (related or unrelated), we checked whether the associations were similar for the intervention and control arms.

Data analysis was conducted with SAS software version 9.4 (SAS Institute, Inc., Cary, NC, USA).

## Results

Almost all participants (96.7%) had a European background. Mean (SD) maternal pre-pregnancy BMI was 23.2 (4.1) kg/m^2^; 26.8% were overweight or obese. Smoking occurred in 11.4% of pregnancies. Mean birth weight of the infants was 3470 (515) g, and mean gestational age was 39.7 (1.6) weeks (Table 1). The percentages boys and girls (~50%) and non-European background (<5%) were similar among studies, but the prevalence of other covariates varied, e.g. smoking during pregnancy varied between 0 and 23.3% (Table 1).

**Table 1:** Characteristics of the studies, and total and cohort-specific descriptive statistics of the study participants

Covariates	Total	COPSAC2000	COPSAC2010	CU Trial	EDEN	KOALA	OCC	PIAMA
Study characteristics								
*N*^a^	2188	278	532	127	721	75	308	147
Year of recruitment	–	2000	2010	1998–1999	2003–2006	2000–2002	2010–2012	1996–1997
Age at outcome measurement, in years	–	12; 18	3; 6; 8	2.5; 7; 13	3; 5	6	0.4;1.5; 3; 5	12; 16
Maternal characteristics								
Age at child’s birth, in years (mean, SD)	30.9 (4.4)	30.2 (4.5)	32.6 (4.3)	30.9 (3.8)	30.0 (4.7)	33.2 (3.6)	30.6 (4.1)	31.2 (3.5)
Low educational level (%, *n*)	12.8 (273)	39.2 (109)	2.3 (12)	2.4 (3)	16.7 (120)	4.0 (3)	4.4 (11)	10.2 (15)
Non-European place of birth (%, *n*)	3.3 (65)	2.9 (8)	4.0 (21)	n.a	3.5 (25)	1.4 (1)	2.4 (6)	3.5 (5)
BMI, in kg/m^2^ (mean, SD)	23.2 (4.1)	n.a	24.1 (4.4)	22.5 (2.7)	22.9 (4.4)	23.5 (3.3)	24.0 (4.3)	22.4 (2.9)
Overweight status Underweight Normal weight Overweight Obese	6.2 (64) 69.0 (1047) 18.0 (273) 6.8 (103)	n.a n.a n.a n.a	1.9 (3) 68.4 (108) 20.9 (33) 8.9 (14)	4.7 (6) 78.7 (100) 16.5 (21) 0 (0)	9.4 (67) 67.8 (483) 15.3 (109) 7.4 (53)	2.7 (2) 69.3 (52) 22.7 (17) 5.3 (4)	3.9 (12) 62.5 (192) 24.1 (74) 9.5 (29)	2.9 (4) 81.2 (112) 13.8 (19) 2.2 (3)
Smoking (%, *n*)	11.4 (241)	23.0 (64)	4.9 (26)	1.8 (2)	19.6 (139)	0.0 (0)	0.3 (1)	7.5 (11)
Gestational age, in weeks (mean, SD)	39.7 (1.6)	40.0 (1.5)	39.9 (1.6)	40.1 (1.1)	39.3 (1.6)	39.6 (1.2)	39.7 (1.4)	40.2 (1.3)
Child characteristics								
Infant’s birth weight, in grams (mean, SD)	3470 (515)	3557 (507)	3560 (530)	3595 (448)	3291 (498)	3593 (462)	3571 (496)	3543 (490)
Infant’s sex (%, *n*) boys	50.5 (1076)	50.7 (141)	49.4 (263)	43.3 (55)	49.7 (358)	51.4 (38)	51.3 (158)	48.3 (7.1)
Breastfeeding duration, in weeks (mean, SD)	31.5 (19.3)	37.9 (21.2)	37.5 (19.0)	37.5 (14.9)	19.5 (15.0)	30.6 (14.9)	44.5 (16.6)	30.9 (12.6)

^a^Children whose mothers provided a breast milk sample and have at least one measurement of blood pressure in childhood

Mean (SD) breast milk DHA level was 0.49 (0.28) wt%. The study-specific mean values of breast milk DHA varied from 0.22 (0.20) to 0.76 (0.47) wt% (Supplementary Table 2). Mean SBP and DBP *z*-scores were 0.49 (0.29) and 0.86 (1.05), respectively. In general, we observed that children at the lower ages had the highest BP *z*-scores. Study-specific averages (SD) of SBP and DBP varied from 93.9 (8.4) to 116.0 (9.9) mmHg, and from 50.7 (7.7) to 71.5 (5.6) mmHg, respectively (Online resource 1; Table 2).

**Fig. 1:**
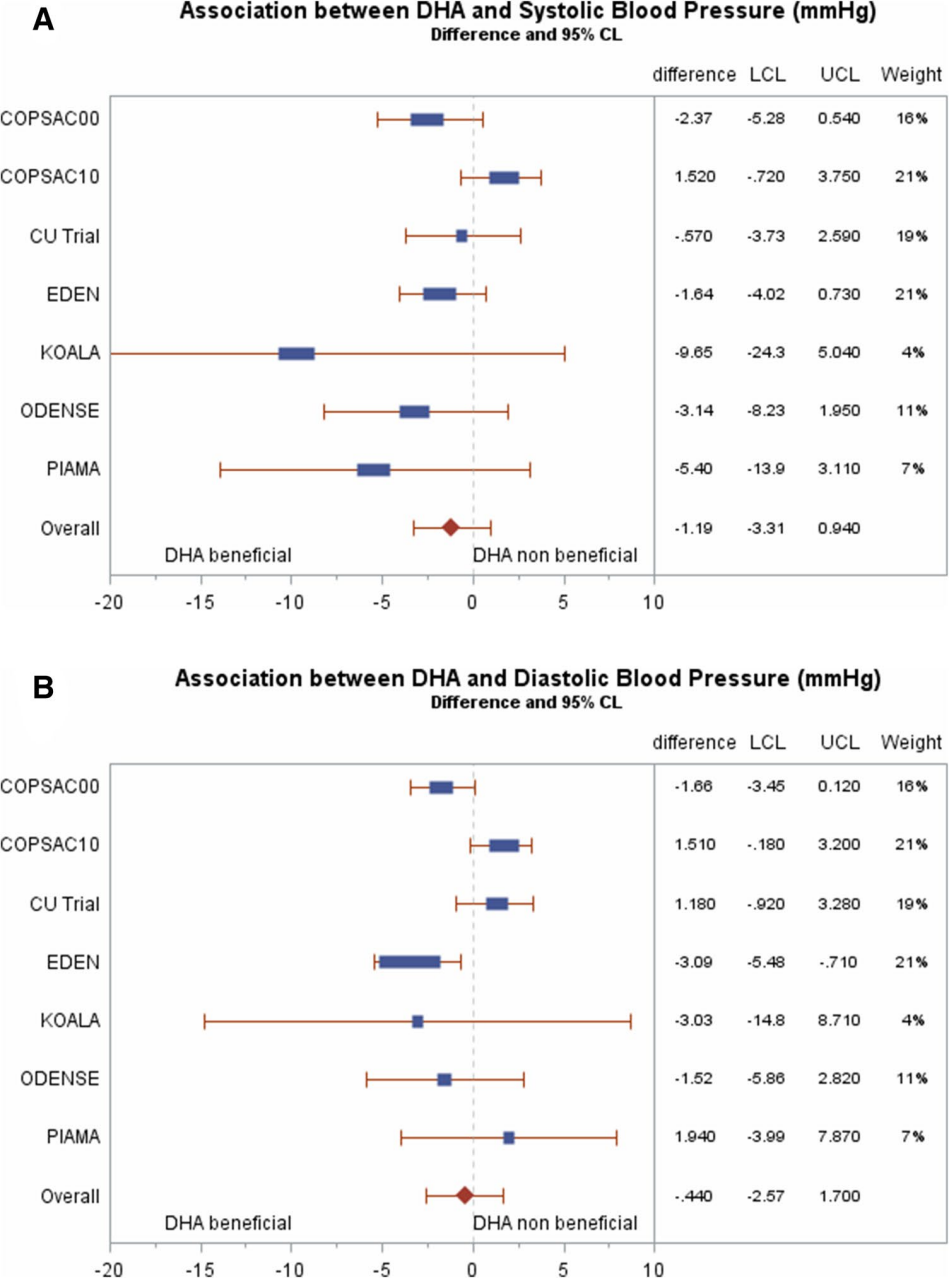
**a** Cohort-specific and pooled mean difference (95% CI) in systolic blood pressure (mmHg) per 0.1 wt% increase in breast milk DHA levels (two-stage meta-analysis). Figure represents numbers of Table 2, model 2. Overall estimate: − 1.19 mmHg (95% CI − 3.31, 0.94), *Q* = 3.45, *df* = 6, *I*^2^ = 0%. **b** Cohort-specific and pooled mean difference (95% CI) in diastolic blood pressure (mmHg) per 0.1 wt% increase in breastmilk DHA levels (two-stage meta-analysis). Figure represents numbers of Table 2, model 2. Overall estimate: − 0.44 mmHg (95% CI − 2.57, 1.70), *Q* = 4.51, *df* = 6, *I*^2^ = 0%

For most studies, the estimate for the association between DHA and SBP was below 0, ranging from − 0.57 to − 9.65 mmHg lower SBP for each 0.1 wt% increase in DHA. Confidence intervals of these estimates included 0 for all studies. For DBP, the estimates ranged from − 3.09 to 1.94 mmHg, with confidence intervals overlapping 0 for all but one study (Table 2). The overall pooled analysis did not show any significant associations as the combined estimate showed that each 0.1 wt% increase in DHA was associated with a − 1.19 (95% CI − 3.31, 0.94) mmHg lower SBP, and a − 0.44 (95% CI − 2.57, 1.70) mmHg lower DBP (Table 3; Fig. 1). Similar results were seen when DHA was dichotomized at the median level of each study (Online resource 1; Supplementary Table 2). There was no association between breast milk DHA and child’s height or between breast milk DHA and child’s BMI (data not shown). For those studies that had an intervention component in the design of the original study (COPSAC2010, CU Trial, PIAMA), the associations were independent of the intervention arm.

**Table 2:** Mean (SD) of breast milk fatty acids and blood pressure for each study participating in the meta-analysis

Acronym	Breast milk fatty acids (wt%); mean (SD)			Blood pressure; mean (SD)				
	DHA	EPA	DHA:AA	Age (years)	Systolic (mmHg)	Systolic (*z*-score)	Diastolic (mmHg)	Diastolic (*z*-score)
COPSAC2000	0.54 (0.29)	0.12 (0.08)	0.92 (0.44)	12 18	111.9 (8.8) 116.0 (9.9)	0.40 (0.79) 0.08 (0.86)	68.7 (5.3) 71.5 (5.6)	0.52 (0.52) 0.37 (0.63)
COPSAC2010	0.36 (0.19)	0.12 (0.07)	1.00 (0.46)	3 6 8	95.8 (6.4) 101.4 (6.3) 103.2 (6.0)	0.55 (0.60) 0.64 (0.62) 0.54 (0.61)	61.8 (4.8) 64.1 (4.9) 65.3 (4.4)	1.37 (0.50) 0.80 (0.52) 0.62 (0.46)
CU Trial	0.76 (0.47)	0.19 (0.12)	1.60 (0.97)	2.5 7 13	110.8 (10.7) 101.9 (6.8) 107.3 (7.3)	1.72 (0.62) 0.53 (0.69) 1.34 (0.74)	66.2 (9.1) 65.6 (5.3) 62.9 (5.7)	1.74 (0.60) 0.77 (0.56) 0.90 (0.90)
EDEN	0.66 (0.22)	0.07 (0.05)	0.77 (0.24)	3 5	93.9 (8.4) 102.0 (7.9)	0.34 (0.79) 0.77 (0.75)	50.7 (7.7) 55.6 (8.3)	0.21 (0.78) 0.05 (0.83)
KOALA	0.41 (0.17)	0.10 (0.05)	0.81 (0.37)	6	105.1 (9.6)	1.03 (0.85)	60.8 (8.1)	0.47 (0.81)
OCC	0.29 (0.17)	0.10 (0.07)	0.82 (0.44)	0.4 1.5 3 5	101.9 (12.6) 102.1 (8.9) 98.2 (7.3) 101.1 (6.5)	1.54 (1.20) 1.28 (0.84) 0.63 (0.69) 0.53 (0.64)	61.6 (10.1) 63.5 (7.6) 62.0 (5.7) 63.7 (6.4)	2.28 (0.89) 1.88 (0.68) 1.20 (0.51) 0.80 (0.57)
PIAMA	0.22 (0.20)	0.06 (0.05)	0.59 (0.54)	12 16	114.5 (9.5) 114.9 (8.7)	0.66 (0.86) 0.18 (0.80)	66.3 (6.5) 65.3 (6.4)	0.31 (0.65) − 0.23 (0.68)

**Table 3:** Cohort-specific and pooled estimates of the associations between breast milk DHA level and blood pressure

	Model 1^a^		Model 2^b^	
	SBP (mmHg)	DBP (mmHg)	SBP (mmHg)	DBP (mmHg)
COPSAC2000	− 1.81 (− 4.67, 1.06)	− 1.75 (− 3.50, − 0.003)	− 2.37 (− 5.28, 0.54)	− 1.66 (− 3.45, 0.12)
COPSAC2010	1.33 (− 0.82, 3.49)	1.25 (− 0.38, 2.88)	1.52 (− 0.72, 3.75)	1.51 (− 0.18, 3.20)
CU Trial	− 1.42 (− 4.11, 1.27)	0.64 (− 1.16, 2.43)	− 0.57 (− 3.73, 2.59)	1.18 (− 0.92, 3.28)
EDEN	− 2.17 (− 4.40, 0.06)	− 3.06 (− 5.32, − 0.80)	− 1.64 (− 4.02, 0.73)	− 3.09 (− 5.48, − 0.71)
KOALA	− 3.83 (− 17.1, 9.45)	3.92(− 7.03, 14.9)	− 9.65 (− 24.3, 5.04)	− 3.03 (− 14.78, 8.71)
OCC	− 2.84 (− 7.27, 1.59)	− 2.01 (− 5.61, 1.58)	− 3.14 (− 8.23, 1.95)	− 1.52 (− 5.86, 2.82)
PIAMA	− 4.26 (− 11.39, 2.87)	3.03 (− 2.05, 8.11)	− 5.40 (− 13.91, 3.11)	1.94 (− 3.99, 7.87)
Pooled	− 1.29 (− 3.21, 0.62)	− 0.42(− 2.45, 1.60)	− 1.19 (− 3.31, 0.94)	− 0.44 (− 2.57, 1.70)

Values are regression coefficients expressed in mmHg for each 0.1 wt% increase in DHA level

*DBP* diastolic blood pressure, *SBP* systolic blood pressure

^a^Adjusted for height, sex, and age

^b^ Model 1 + maternal smoking, maternal educational level, maternal age, maternal BMI (not for COPSAC2000/COPSAC2010), gestational age, child’s birth weight, maternal place of birth (not for CU Trial), and breastfeeding duration

**Fig. 2:**
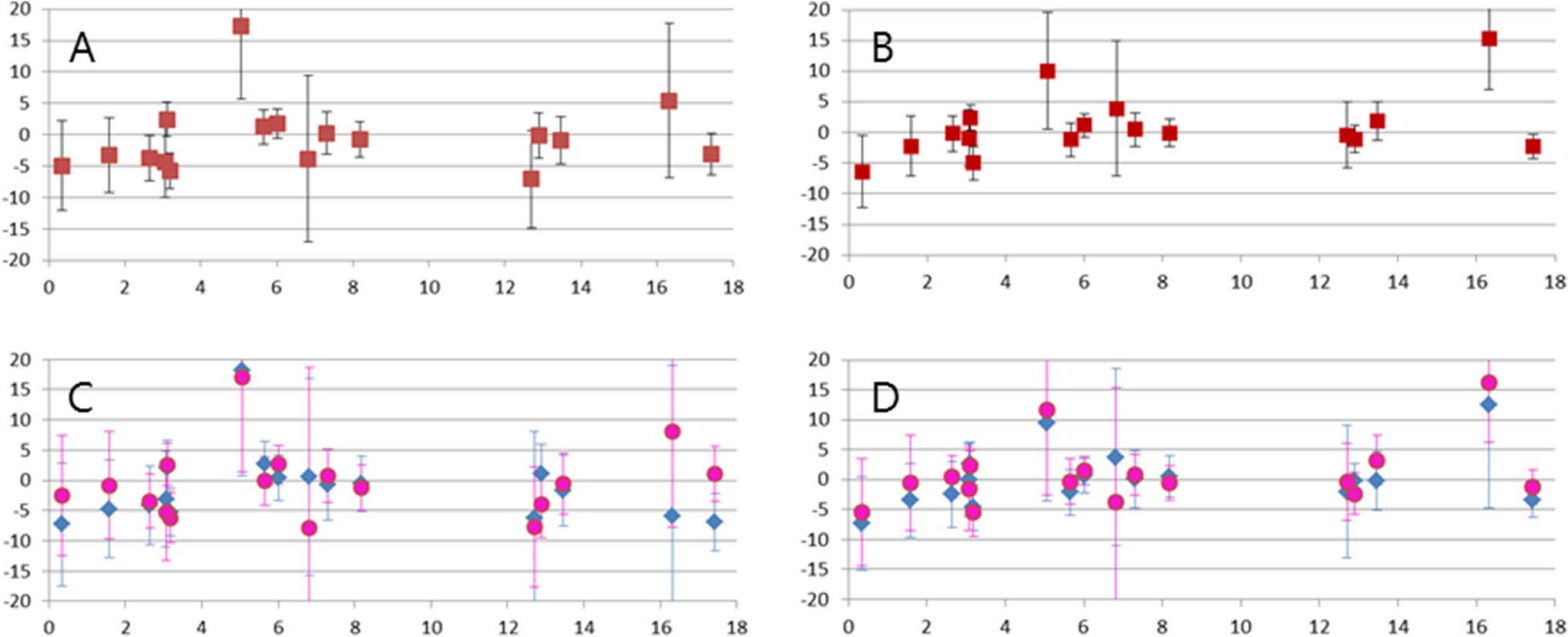
Difference in systolic (**a**, **c**) and diastolic (**b**, **d**) blood pressure (mmHg) per 0.1 wt% increase in breastmilk DHA levels, displayed for increasing age (years), and stratified by sex (**c**, **d**; pink bullets are girls, blue diamonds are boys)

For SBP, the *p* value for the interaction term for DHA × age was <0.10 in 4 out of 7 studies; for DBP this was 3 out of 7 studies. There was no clear age-related pattern for the association between DHA and BP (Supplementary Table 3 of online resource 1 and illustrated in Fig. 2a, c). The *p* value for the interaction term for DHA × sex was >0.10 in all studies, but the interaction term for DHA × age × sex had a *p* value <0.10 in 1 out of 7 studies for SBP and DBP. In general, the estimates for boys and girls across ages seems to be close from each other at all ages, but tended to diverge for SBP in early life and adolescence, where blood pressure was lower for boys than girls per wt% increase in breast milk DHA (Fig. 2b, d).

Overall, there were no associations between EPA and the ratio of DHA/AA and BP (systolic or diastolic) (Supplementary Tables 4 and 5 of online resource 1, respectively).

## Discussion

### Summary of main findings

This individual participant meta-analysis of seven studies representing 2188 children with BP assessment in the full range from infancy to adolescence, observed no overall association between systolic or DBP and increasing levels of breast milk DHA ranging from mean of 0.22–0.76 wt%. There were also no associations between BP and increasing levels of EPA or of DHA/AA ratio in breast milk, and no signs of age or sex differences in the associations.

### Comparison with earlier studies and implications

Our result is in line with four earlier publications that reported no association between early life n-3 LC PUFAs intake and BP in childhood [17, 19, 21, 23], with an age at outcome measurement ranging from 2.5 to 9 years. These studies directly supplemented infants (280 mg/day DHA as ethyl-ester form from birth to 6 months) [19] or infant formula with n-3 LC PUFA (0.30 wt% DHA as triglyceride and phospholipid forms from birth to 2 months) [17], and one of the studies [21, 23] supplemented lactating mothers with fish oil. Conversely, two other studies reported that n-3 LC PUFAs were associated with a lower BP [16, 18]. One of which was a multicentric European RCT that supplemented infant formula with 0.15–0.25 wt% of DHA (as egg phospholipid form) found a lower BP in 6-year-old children (− 3.6 mmHg for DBP; not significant for SBP) [18]. The second one was our previous observational study, where we found that children who received breast milk with a DHA ≥ 0.18 wt% had a lower BP (− 4.79 mmHg for SBP and − 2.47 mmHg for SBP) at the age of 12 compared to children who received infant formula with no DHA (with adjustment for child’s sex) [16]. Another report found a lower BP with higher mother’s milk n-3 LCPUFAs (ranging 0.24–0.47 wt%, with 75% being DHA) but only in boys (SBP and DBP) at 4 months of age, then only in girls (SBP) at 18 months of age, and no association regardless the infant sex at 36 months of age [43]. One study found a higher BP in boys whose mothers were supplemented with fish oil during lactation at the age of 7 years [20], with replication of the findings at the age of 13 [22]. Our meta-analysis did not show clear sex differences for the association between DHA and BP, though the associations seemed to be more beneficial for boys, especially in early age and adolescence. The differences in the associations between n-3 LC PUFA and BP in the earlier studies cited herein does not seem to be related to study design, age at BP assessment, method of administration, or level of n-3 LC PUFA. This meta-analysis adds to the literature by including data from several unpublished studies with data on breast milk DHA, and by harmonizing the data to enhance comparability. Relevant issues to consider in future studies are potential interference from other components in infant feeding or lifestyle factors, the latter being important to study in the context of an RCT design.

### Methodological considerations

The strength of our study is that we included all eligible studies, irrespective of whether these studies had published the results. Moreover, most studies did not have the association of interest in this paper as a primary research question, which prevented publication bias [44]. Also, the studies included in the meta-analysis had a wide range of breast milk DHA, and our associations represent increments that can be expected by supplementing women with the recommended dose of DHA [45, 46]. However, our study has some limitations that should be taken into account when interpreting the results. First, all cohorts had loss-to-follow-up in the period between breast milk collection and BP measurements. This could have led to selection bias if those with a low or high DHA level and a low or high BP were selectively lost to follow up. Second, when converting the BPs to *z*-scores by the percentiles of “The Fourth Report on the Diagnosis, Evaluation, and Treatment of High BP in Children and Adolescents”, we noticed relatively high BP *z*-scores in all studies included in this meta-analysis, but especially in younger children. This is possibly related to the method of measurements (e.g. number of repeats) as well as the difficulty of measuring BP in young children. Although this would not have affected validity, associations between DHA and BP could be less precise in younger children. Indeed, absolute BPs were comparable with an earlier study investigating the association between DHA and BP in children, but SEs were smaller in this study, and statistically significant associations were detected [42]. Third, most studies used a single sample of breast milk to determine the amount of breast milk n-3 LCPUFAs, which may have led to misclassification of the exposure as breastmilk n-3 LCPUFA vary from day to day based on the mothers recent intake of fish [47]. Thus, the latter two limitations may have prevented us to find a real association between n-3 LC PUFAs and BP, if present.

### Clinical relevance

The results reported in our study are per 0.1 wt% increase in breast milk fatty acids, which is a relatively small increase, and results in a small potential change in BP which is not clinically relevant. Though statistical significance and precision remains unchanged, we illustrate implications for clinical relevant changes in BP if breast milk DHA increases to a larger, but feasible, extent. Per SD increase in DHA, DHA is associated with a 3.24 mmHg lower (95% CI − 8.59, 2.10) SBP, and a 1.11 mmHg lower (95% CI − 5.68, 3.46) DBP. If breast milk DHA increases with 0.8 wt% (which is the increase that was measured after supplementing women with fish oil during lactation), DHA is associated with a 9.52 mmHg lower (95% CI − 26.51, 7.47) SBP, and a 3.50 mmHg lower (95% CI − 20.59, 13.58) DBP.

### Implications

Although hypertension is rare in childhood [48], BP tends to track from childhood to adulthood from a very young age [49]. Therefore, the less increased the BP in childhood, the lower the risk of hypertension in adulthood, which implies that prevention of hypertension should start early in life. Our results do not support a beneficial effect of breast milk n-3 LC PUFAs on childhood BP. However, the current global recommendations for pregnant and lactating women of 200 mg/day of DHA intake (obtained for example by one portion of oily fish a week) still apply for other health outcomes [50].

## Conclusion

This meta-analysis does not support an association between breast milk n-3 LC PUFAs and BP throughout childhood in boys or girls.

## Electronic supplementary material

Below is the link to the electronic supplementary material.

Supplementary file1 (DOCX 40 kb)
