# Supplementary material for: Breast milk n-3 long-chain polyunsaturated fatty acids and blood pressure: an individual participant meta-analysis
Source: Eur J Nutr. 2020 Jun 20;60(2):989–98. doi: 10.1007/s00394-020-02310-4 (PMC7900030; doi:10.1007/s00394-020-02310-4)
Supplement: Supplementary file 1 — Supplementary file1 (DOCX 40 kb) [file 394_2020_2310_MOESM1_ESM.docx]

**Supplementary Material**

**Breast milk fatty acids and blood pressure in childhood. An individual participant data meta-analysis.**

Table of contents:

Supplementary Table 1: Methods of blood pressure measurements and fatty acids analysis

Supplementary Table 2: Difference (95% CI) in blood pressure between high and low levels (median split) of breast milk DHA, for each study

Supplementary Table 3: Difference (95% CI) in blood pressure per 0.1wt% increase in breast milk DHA, estimates for each age separately in cohorts with repeated outcome measurements.

Supplementary Table 4: Cohort-specific and pooled estimates of the associations between breast milk EPA level and blood pressure.

Supplementary Table 5: Cohort-specific and pooled estimates of the associations between breast milk DHA:AA ratio and blood pressure.

**Supplementary Table 1: Methods of fatty acids analysis and blood pressure measurements**

| **Acronym** | **Measurement of breast milk fatty acids** | | | **Measurement of blood pressure** |
| --- | --- | --- | --- | --- |
|  | Reference | Sample extraction/ derivatization | Milk Sample | Device |
| COPSAC2000[1] | [2] | Bligh and Dyer/KOH in methanol | 1 mo postpartum | Welch Allyn, ProBP3400 |
| COPSAC2010[3] | [4] | Folch/BF_3_ in methanol | 1 mo postpartum | Welch Allyn, ProBP3400 |
| CU Trial[5] | [5] | Bligh and Dyer/KOH in methanol | 1 wk, 2 mo, 4 mo postpartum | Boso-medicus Prestige unit (Bosch + Sohn) |
| EDEN[6] | [7] | Direct transesterification w/ acetyl chloride | Colostrum 2-5d | COLIN BP-8800 C |
| KOALA[8] | [9] | Folch/KOH in methanol | 1 mo postpartum | Omron 705IT |
| OCC[10] | [11] | Folch/BF_3_ in methanol | Fat fraction  4mo postpartum | Welch Allyn Connex^®^ Vital Signs Monitor 6000 Series™ |
| PIAMA[12] | [13] | Direct transesterification w/ methanol HCL | 2-35 wks postpartum | Omron M6 |

**Supplementary Table 2: Difference (95% CI) in blood pressure between high and low levels (median split) of breast milk DHA, for each study**

|  | Median split (wt%) | Difference (low=reference group) in blood pressure in mmHg (95% CI)^a^ | |
| --- | --- | --- | --- |
|  |  | Systolic | Diastolic |
| COPSAC2000 | 0.47 | -1.70 (-3.53, 0.13) | -0.66 (-1.78, 0.46) |
| COPSAC2010 | 0.32 | -0.12 (-0.96, 0.72) | 0.19 (-0.44, 0.83) |
| CU Trial | 0.62 | -1.44 (-3.98, 1.10) | -0.16 (-1.86, 1.55) |
| EDEN | 0.62 | -0.17 (-1.16, 0.83) | -0.61 (-1.62, 0.40) |
| KOALA | 0.38 | -3.82 (-8.33, 0.68) | 0.28 (-3.52, 4.07) |
| OCC | 0.25 | -0.31 (-1.83, 1.22) | 0.02 (-1.21, 1.25) |
| PIAMA | 0.17 | -2.32 (-5.28, 0.63) | 0.08 (-2.06, 2.21) |

^a^ Adjusted for age, sex, and height

**Supplementary Table 3: Difference (95% CI)^a^ in blood pressure per 0.1wt% increase in breast milk DHA, estimates for each age separately in cohorts with repeated outcome measurements.**

|  | **SBP (mmHg)** | **DBP (mmHg)** |
| --- | --- | --- |
| Study |  |  |
| COPSAC2000  *age 12*  *age 18* | -0.15 (-3.74, 3.44)  -3.04 (-6.34, 0.26) | -1.06 (-3.28, 1.15)  -2.27 (-4.30, -0.24) |
| COPSAC2010  *age 3*  *age 6*  *age 8* | 2.46 (-0.25, 5.18)  1.74 (-0.66, 4.14)  -0.76 (-3.64, 2.11) | 2.39 (0.29, 4.49)  1.12 (-0.72, 2.95)  -0.06 (-2.30, 2.17) |
| CU Trial  *age 2.5*  *age 7*  *age 13* | -3.67 (-7.26, -0.07)  0.25 (-3.16, 3.67)  -0.94 (-4.71, 2.82) | -0.20 (-3.15, 2.75)  0.46 (-2.27, 3.20)  1.82 (-1.31, 4.94) |
| EDEN  *age 3*  *age 5* | -5.76 (-8.57, -2.94)  1.25 (-1.51, 4.02) | -5.00 (-7.75, -2.25)  -1.21 (-3.92, 1.50) |
| OCC  *3 months*  *18 months*  *3 years*  *5 years* | -4.93 (-12.04, 2.18)  -3.20 (-9.14, 2.74)  -4.37 (-9.93, 1.20)  17.23 (5.60, 28.87) | -6.37 (-12.20, -0.53)  -2.25 (-7.12, 2.62)  -0.93 (-5.49, 3.63)  10.05 (0.50, 19.60) |
| PIAMA  *age 12*  *age 16* | -7.09 (-14.79, 0.61)  5.42 (-6.92, 17.77) | -0.41 (-5.77, 4.96)  15.33 (7.00, 23.65) |

^a^ Adjusted for age, height, and sex

**Supplementary Table 4: Cohort-specific and pooled estimates of the associations between breast milk EPA level and blood pressure.**

|  | **Model 1^a^** |  | **Model 2**^b^ |  |
| --- | --- | --- | --- | --- |
|  | SBP (mmHg) | DBP (mmHg) | SBP (mmHg) | DBP (mmHg) |
| COPSAC2000 | -9.07 (-19.82, 1.68) | -7.56 (-14.13, -1.00) | -10.96 (-21.86, -0.06) | -7.30 (-13.99, -0.60) |
| COPSAC2010 | 4.38 (-1.43, 10.18) | 3.82 (-0.55, 8.20) | 3.88 (-2.00, 9.76) | 3.51 (-0.94, 7.95) |
| CU Trial | -7.22 (-18.31, 3.86) | 2.50 (-4.96, 9.95) | -3.75 (-16.43, 8.93) | 4.55 (-3.96, 13.05) |
| EDEN | -10.98 (-22.07, 0.11) | -9.09 (-20.33, 2.15) | -10.87 (-22.35, 0.60) | -11.30 (-22.83, 0.22) |
| KOALA | -6.16 (-52.25, 7.86) | 8.78 (-29.25, 46.80) | -20.48 (-69.78, 28.83) | -4.54 (-43.69, 34.61) |
| OCC | -3.69 (-15.25, 7.86) | 2.94 (-6.40, 12.28) | -6.04 (-21.51, 9.43) | 5.95 (-7.16, 19.07) |
| PIAMA | -21.09 (-47.05, 4.86) | 6.24 (-12.40, 24.87) | -17.61 (-47.07, 11.85) | 5.91 (-14.61, 26.42) |
|  |  |  |  |  |
| Pooled | -5.27 (-13.21, 2.67) | -0.25 (-6.43, 5.93) | -5.44 (-13.83, 2.95) | -0.39 (-7.59, 6.80) |

Values are regression coefficients expressed in mmHg for each 0.1wt% increase in DHA level.

^a^ Adjusted for height, sex, and age

^b^ Model 1 + maternal smoking, maternal educational level, maternal age, maternal BMI (not for COPSAC2000 / COPSAC2010), gestational age, child’s birth weight, maternal place of birth (not for CU Trial), and breastfeeding duration.

Abbreviations: DBP, diastolic blood pressure; SBP, systolic blood pressure.

**Supplementary Table 5: Cohort-specific and pooled estimates of the associations between breast milk DHA/AA ratio and blood pressure.**

|  | **Model 1**^a^ |  | **Model 2**^b^ |  |
| --- | --- | --- | --- | --- |
|  | SBP (mmHg) | DBP (mmHg) | SBP (mmHg) | DBP (mmHg) |
| COPSAC2000 | -0.48 (-2.34, 1.37) | -0.76 (-1.89, 0.38) | -0.87 (-2.76, 1.01) | -0.75 (-1.90, 0.41) |
| COPSAC2010 | 0.74 (-0.16, 1.65) | 0.72 (0.04, 1.41) | 0.76 (-0.17, 1.68) | 0.73 (0.03, 1.43) |
| CU Trial | -0.24 (-1.56, 1.08) | 0.44 (-0.43, 1.31) | 0.16 (-1.31, 1.63) | 0.65 (-0.32, 1.62) |
| EDEN | -1.95 (-4.06, 0.15) | -2.76 (-4.89, -0.62) | -1.38 (-3.64, 0.89) | -3.13 (-5.41, -0.86) |
| KOALA | -0.87 (-7.09, 5.35) | 1.94 (-3.17, 7.06) | -4.89 (-12.18, 2.40) | -2.53 (-8.34, 3.28) |
| OCC | -1.29 (-2.98, 0.41) | -0.85 (-2.23, 0.52) | -1.53 (-3.50. 0.45) | -0.99 (-2.68, 0.69( |
| PIAMA | -1.62 (-4.27, 1.04) | 0.85 (-1.04, 2.75) | -1.79 (-4.87, 1.28) | 0.51 (-1.63, 2.64) |
|  |  |  |  |  |
| Pooled | -0.52 (-1.64, 0.59) | -0.14 (-1.21, 0.93) | -0.51 (-1.71, 0.69) | -0.33 (-1.57, 0.90) |

Values are regression coefficients expressed in mmHg for an increase of one point in the DHA/AA ratio.

^a^ Adjusted for height, sex, and age

^b^ Model 1 + maternal smoking, maternal educational level, maternal age, maternal BMI (not for COPSAC2000 / COPSAC2010), gestational age, child’s birth weight, maternal place of birth (not for CU Trial), and breastfeeding duration.

Abbreviations: DBP, diastolic blood pressure; SBP, systolic blood pressure.

**References**

[1] Bisgaard H (2004) The Copenhagen Prospective Study on Asthma in Childhood (COPSAC): design, rationale, and baseline data from a longitudinal birth cohort study. Ann Allergy Asthma Immunol 93 (4):381-389. doi:S1081-1206(10)61398-1 [pii];10.1016/S1081-1206(10)61398-1 [doi]

[2] Lauritzen L, Halkjaer LB, Mikkelsen TB, et al. Fatty acid composition of human milk in atopic Danish mothers. Am J Clin Nutr 2006; 84(1):190-196.

[3] Bisgaard H, Vissing NH, Carson CG, Bischoff AL, Folsgaard NV, Kreiner-Moller E, Chawes BL, Stokholm J, Pedersen L, Bjarnadottir E, Thysen AH, Nilsson E, Mortensen LJ, Olsen SF, Schjorring S, Krogfelt KA, Lauritzen L, Brix S, Bonnelykke K (2013) Deep phenotyping of the unselected COPSAC2010 birth cohort study. Clin Exp Allergy 43 (12):1384-1394. doi:10.1111/cea.12213 [doi]

[4] Bisgaard H, Stokholm J, Chawes BL, et al, Thorsteinsdottir S, Folsgaard NV, Fink NR, Thorsen J, Pedersen AG, Waage J, Rasmussen MA, Stark KD, Olsen SF, Bonnelykke K. Fish Oil-Derived Fatty Acids in Pregnancy and Wheeze and Asthma in Offspring. N Engl J Med 2016; 375(26):2530-2539.

[5] Lauritzen L, Jorgensen MH, Mikkelsen TB, Skovgaard l, Straarup EM, Olsen SF, Hoy CE, Michaelsen KF (2004) Maternal fish oil supplementation in lactation: effect on visual acuity and n-3 fatty acid content of infant erythrocytes. Lipids 39 (3):195-206

[6] Heude B, Forhan A, Slama R, Douhaud L, Bedel S, Saurel-Cubizolles MJ, Hankard R, Thiebaugeorges O, De AM, Annesi-Maesano I, Kaminski M, Charles MA (2016) Cohort Profile: The EDEN mother-child cohort on the prenatal and early postnatal determinants of child health and development. Int J Epidemiol 45 (2):353-363. doi:dyv151 [pii];10.1093/ije/dyv151 [doi]

[7] Armand M, Bernard JY, Forhan A, Heude B, Charles MA (2018) Maternal nutritional determinants of colostrum fatty acids in the EDEN mother-child cohort. Clin Nutr 37 (6 Pt A):2127-2136. doi:S0261-5614(17)31367-5 [pii];10.1016/j.clnu.2017.10.007 [doi]

[8] Kummeling I, Thijs C, Penders J, Snijders BE, Stelma F, Reimerink J, Koopmans M, Dagnelie PC, Huber M, Jansen MC, de BR, van den Brandt PA (2005) Etiology of atopy in infancy: the KOALA Birth Cohort Study. Pediatr Allergy Immunol 16 (8):679-684. doi:PAI333 [pii];10.1111/j.1399-3038.2005.00333.x [doi]

[9] Thijs C, Muller A, Rist L, et al. Fatty acids in breast milk and development of atopic eczema and allergic sensitisation in infancy. Allergy 2011; 66(1):58-67.

[10] Kyhl HB, Jensen TK, Barington T, Buhl S, Norberg LA, Jorgensen JS, Jensen DF, Christesen HT, Lamont RF, Husby S (2015) The Odense Child Cohort: aims, design, and cohort profile. Paediatr Perinat Epidemiol 29 (3):250-258. doi:10.1111/ppe.12183 [doi]

[11] Bruun S, van Rossem L, Lauritzen L, Husby S, Neergaard JL, Michaelsen KF, Boysen SM, Stark KD, Sorensen J, Zachariassen G (2019) Content of n-3 LC-PUFA in Breast Milk Four Months Postpartum is Associated with Infancy Blood Pressure in Boys and Infancy Blood Lipid Profile in Girls. Nutrients 11 (2). doi:nu11020235 [pii];10.3390/nu11020235 [doi]

[12] Wijga AH, Kerkhof M, Gehring U, de Jongste JC, Postma DS, Aalberse RC, Wolse AP, Koppelman GH, van RL, Oldenwening M, Brunekreef B, Smit HA (2014) Cohort profile: the prevention and incidence of asthma and mite allergy (PIAMA) birth cohort. Int J Epidemiol 43 (2):527-535. doi:dys231 [pii];10.1093/ije/dys231 [doi]

[13] Wijga A, Houwelingen AC, Smit HA, Kerkhof M, Vos AP, Neijens HJ, Brunekreef B. Fatty acids in breast milk of allergic and non-allergic mothers: The PIAMA birth cohort study. Pediatr Allergy Immunol 2003; 14(3):156-162.
